# Diagnostic value of gadolinium contrast administration for spinal cord magnetic resonance imaging in multiple sclerosis patients and correlative markers of lesion enhancement

**DOI:** 10.1177/20552173211047978

**Published:** 2021-11-26

**Authors:** Kianush Karimian-Jazi, Ulf Neuberger, Katharina Schregel, Gianluca Brugnara, Daniel Schwarz, Laura Bettina Jäger, Wolfgang Wick, Martin Bendszus, Michael O. Breckwoldt

**Affiliations:** Neurology Clinic, University Hospital Heidelberg, Heidelberg, Germany; ^1^Clinical Cooperation Unit Neurooncology, German Cancer Consortium (DKTK) within the German Cancer Research Center (DKFZ), Heidelberg, Germany ^2^Neurology Clinic and National Center for Tumor Diseases, University Hospital Heidelberg, Heidelberg, Germany; Department of Neuroradiology, University Hospital Heidelberg, Heidelberg, Germany

**Keywords:** Multiple sclerosis, spinal magnetic resonance imaging, gadolinium-based contrast agents, T2 signal ratio, gadolinium depositions

## Abstract

**Background:**

Magnetic resonance imaging is essential for monitoring people with multiple sclerosis, but the diagnostic value of gadolinium contrast administration in spine magnetic resonance imaging is unclear.

**Objective:**

To assess the diagnostic value of gadolinium contrast administration in spine magnetic resonance imaging follow-up examinations and identify imaging markers correlating with lesion enhancement.

**Methods:**

A total of 65 multiple sclerosis patients with at least 2 spinal magnetic resonance imaging follow-up examinations were included. Spine magnetic resonance imaging was performed at 3 Tesla with a standardized protocol (sagittal and axial T2-weighted turbo spin echo and T1-weighted post-contrast sequences). T2 lesion load and enhancing lesions were assessed by two independent neuroradiologists for lesion size, localization, and T2 signal ratio (T2 signal_lesion_/T2 signal_normal appearing spinal cord_).

**Results:**

A total of 68 new spinal T2 lesions and 20 new contrast-enhancing lesions developed during follow-up. All enhancing lesions had a discernable correlate as a new T2 lesion. Lesion enhancement correlated with a higher T2 signal ratio compared to non-enhancing lesions (T2 signal ratio: 2.0 ± 0.4 vs. 1.4 ± 0.2, ****p < 0.001). Receiver operating characteristics analysis showed an optimal cutoff value of signal ratio 1.78 to predict lesion enhancement (82% sensitivity and 97% specificity).

**Conclusion:**

Gadolinium contrast administration is dispensable in follow-up spine magnetic resonance imaging if no new T2 lesions are present. Probability of enhancement correlates with the T2 signal ratio.

## Introduction

Magnetic resonance imaging (MRI) is essential for the initial diagnosis and monitoring of people with multiple sclerosis (MS).^1,2^ Spinal lesions are often associated with coexisting cerebral lesions^3^ and are a known predictor of disease severity.^4–8^ Similarly to cerebral MRI, gadolinium-based contrast agents (GBCAs) are routinely used in follow-up spinal MRI to detect gadolinium (Gd) contrast enhancement (CE) as a marker of acute inflammation.^9^ Recently, there have been major safety concerns regarding Gd accumulation in the brain after multiple injections of linear GBCAs.^10–14^ As a consequence, assessing the additional diagnostic value of Gd administration for all indications is warranted. Recent reports on cerebral follow-up MRI examinations of people with MS demonstrated that Gd contrast administration might be dispensable if the T2 lesion load is stable compared to the preceding examination.^15,16^ In these studies, only a small fraction of lesions (<5%) was identified for which Gd administration added diagnostic value. These were cases of lesion reactivation, persistent enhancement over several months, or CE lesions that were not discernable on T2-weighted (T2-w) imaging. Previous studies have focused on cerebral MRI, whereas the diagnostic value of Gd administration for spinal MRI has not been evaluated. It would be conceivable that the importance of Gd administration in spinal MRI is higher compared to cerebral MRI given the small anatomical structure of the spinal cord with a worse signal-to-noise ratio, artifact-prone scanning, and fewer MR sequences that are acquired in clinical practice. Also, T2 lesion detection in the spinal cord can be challenging, especially for small and lateral cord lesions due to partial volume effects.^17,18^

The present study investigated whether the routine administration of GBCAs is indicated in all follow-up spinal examinations. Moreover, we investigated whether the lesion morphology and signal properties quantified as lesion size and T2 signal ratio (SR) (T2 signal_lesion_/T2 signal_normal appearing spinal cord_) predict CE. Finally, we compared spinal and cerebral examinations in each patient to evaluate if Gd-administration may be justified for spinal MRI if there is CE on cerebral MRI and vice versa.

## Methods

### Patients and study design

We included 65 patients (32 men and 33 women; mean age: 40.6 ± 11.8 years, range: 18–66 years) with confirmed MS and at least 2 spinal MRI follow-up examinations (mean follow-up time: 4.6 ± 2.4 years; a total of 192 spinal MRI examinations) in this retrospective study. All MRI investigations were performed at the Department of Neuroradiology (University Hospital Heidelberg) between 2010 and 2020. The study population consisted of patients with relapsing–remitting (RR) MS (n = 58; 89%), primary progressive MS (n = 1; 2%), and secondary progressive MS (n = 6; 9%). The mean Expanded Disability Status Scale (EDSS) score was 3.1 ± 2.8 and the median disease duration was 6.9 ± 7.4 years. Patients were included in this analysis if the following inclusion criteria were met: confirmed diagnosis of MS, in-house standardized MRI protocol available, and age ≥18 years. As this is a retrospective study, there were no formal exclusion criteria.

### Standard protocol approvals, registrations, and patient consents

Informed consent was obtained from all study participants. The study adhered to the Declaration of Helsinki and was approved by the local ethics committee of the Medical Faculty, University of Heidelberg (study permit number: S-424/2012).

### Spine MRI protocol

MRI was performed on a 3 Tesla MRI scanner (MAGNETOM Skyra, MAGNETOM Verio, MAGNETOM Prisma or Trio, Siemens Healthineers, Erlangen, Germany) and patients were examined in yearly intervals for routine clinical follow-up. A standardized study protocol was used in all patients, including sagittal and axial T2-w and T1-weighted (T1-w) turbo spin echo (TSE) sequences after GBCA administration (0.1 mmol/kg; Clariscan©, GE Healthcare or Dotarem©, Guerbet). T2-w sequences were acquired before contrast administration. Axial T1- and T2-w sequences were performed on the cervical spinal cord to thoracic vertebra 2–3 and additionally at levels of T2 lesions that were identified on the sagittal plane (for further sequence details, see Table 1).

**Table 1.** Magnetic resonance imaging (MRI) sequence parameters.

Parameter	T2 (TSE)	T2 (TSE)	T1 (TSE)	T1 (TSE)
**Orientation**	Axial 2D	Sagittal 2D	Axial 2D	Sagittal 2D
**TE**	102	104	11	9.3
**TR**	4250	3000	600	935
**Flip angle**	150	160	150	125
**FOV**	200 × 200	320 × 320	320 × 320	180 × 180
**Matrix size**	256 × 256	320 × 320	307 × 384	256 × 256
**Slice thickness (mm)**	3	3	3	3
**No. of averages**	2	2	1	2
**In-plane resolution (mm^3^)**	0.8 × 0.8 × 3	0.9 × 0.9 × 3	0.7 × 0.7 × 3	0.9 × 0.9 × 3
**Duration (min:sec)**	4:51	3:32	2:11	6:03

FOV: field of view; TE: echo time; TR: repetition time; TSE: turbo spin echo.

### Cerebral MRI protocol

Study sequences included a fluid-attenuated inversion recovery (FLAIR) sequence, axial T2-w, and T1-w magnetization-prepared rapid acquisition gradient-echo (MP-RAGE) sequence after Gd contrast administration (0.1 mmol/kg; Dotarem, Guerbet, France) as described previously.^15^ T2-w and FLAIR sequences were acquired before contrast administration. We analyzed matched cerebral MRI and spinal MRI investigations to understand patterns of disease activity (379 cerebral and 379 matched spinal scans of the same patient; 758 MRI scans in total).

### Analysis of MRI images

Image analysis was performed by two independent neuroradiologists (K.K.-J. and M.O.B.). First, the number of new T2-w lesions was assessed in comparison with the previous scan on all available, longitudinal MRI follow-up studies. Each reading was compared with the previous MRI scan to detect new T2 lesions. In the second independent reading that was performed blinded to the T2 readings, Gd CE on T1-w images was assessed on all scans. We then compared new T2 lesions with contrast-enhancing lesions after Gd administration to determine which T2 lesions showed enhancement on T1-w CE images and to evaluate possible T2-negative, Gd-enhancing lesions.

“Missed lesion” was defined as a lesion that showed Gd enhancement but was not discernable on T2 images. Ring-enhancement was defined as a ring-shaped Gd enhancement surrounding a central zone of non-enhancing myelon on T1-w post-contrast images. The T2-w signal intensity ratio of CE versus non-enhancing lesions was quantified as T2 signal intensity_lesion_/T2 signal intensity_normal appearing spinal cord_. We also measured the maximum axial area of each new T2 lesion and CE T1 lesion on a single imaging plane and annotated the respective level of the lesion. If the lesion was present on multiple levels the largest lesion area was quantified. Spinal and cerebral imaging results were compared to each other in a time period of ± 3 months to correlate disease activity at each site and calculate an odds ratio (OR) of disease activity.

### Statistical analyses

Data are shown as boxplots with whiskers, mean ±  SD, or median ± SD (as indicated). Statistical analyses were performed in Prism (GraphPad, version 8.4.3) and R version 4.0.0, R core team, (Vienna, Austria). Distribution of data were tested with the Shapiro–Wilk test. Two-tailed Student’s *t*-tests were used to compare two groups for parametrically distributed data. Multiple groups were compared and adjusted *p*-values derived from Tukey’s test following one-way ANOVA are reported. Correlation analysis was performed by calculating Pearson's correlation coefficient. Optimal thresholds to discern contrast-enhancing lesions were calculated using receiver operating characteristic (ROC) curves by maximizing Youden's index and running 1000 bootstrap repeats for the ratio of T2 hyperintensities of spinal lesions and unaffected spinal tissue, which were manually derived from a region-of-interest analysis. Logistic regression analysis was performed and results are given in crude OR and their 95% confidence interval (CI). *p* < 0.05 was considered statistically significant (*p < 0.05; **p < 0.01; ***p < 0.001, ****p < 0.0001).

## Results

### Study characteristics and patient cohort

We included 65 MS patients with a mean follow-up time of 4.6 ± 2.4 years and a median of 3 MRI scans per patient (range: 2–10) in this retrospective study (for patients’ details, see Table 2). In these patients, we identified a total of 228T2 lesions (mean number of lesions per patient at baseline: 3.51 ± 2.41). A total of 68 of these T2 lesions occurred newly during follow-up investigations (mean number of lesions per patient: 3.82 ± 1.84; 52 new lesions in the cervical cord and 16 in the thoracic cord). New T2 lesions were present in 23% of the follow-up scans (44 of 192 MRI scans). Moreover, 20 Gd CE lesions developed during follow-up. All of these were clearly discernible as a new T2 lesion. There was no case of lesion reactivation (i.e. intermittent CE in persistent T2 lesions) or persistent lesion enhancement. As previously reported,^17,19^ most of the new T2 and CE T1 lesions were located in the cervical spinal cord (73% and 75%, respectively), followed by lesions in the thoracic cord (27% and 25%, respectively).

**Table 2.** Clinical characteristics of study patients.

**Total number of included MS patients**	65
**RR**	58
**SP**	6
**PP**	1
**Age**	40.6 + /−11.8 (18–66)
**Male:female**	32:33
**EDSS**	3.0 (1.6−4.0)
**Disease duration (years)**	6.9 + /−7.4 (0−37)
**Follow-up time (years)**	4.6 + /−2.4 (0.3−10.9)
**No. of follow-up MRIs**	3 (2−10)

EDSS: Expanded Disability Status Scale; PP: primary progressive MS; MRI: magnetic resonance imaging; MS: multiple sclerosis; RR: relapsing–remitting MS; SP: secondary progressive MS. Values are shown as mean ± SD (range) for age and disease duration (years after first symptoms of MS) and median (range) for follow-up MRI. Values are shown as median (and interquartile range: 25th–75th percentiles) for EDSS.

### Correlative markers of Gd enhancement

**Figure 1.**
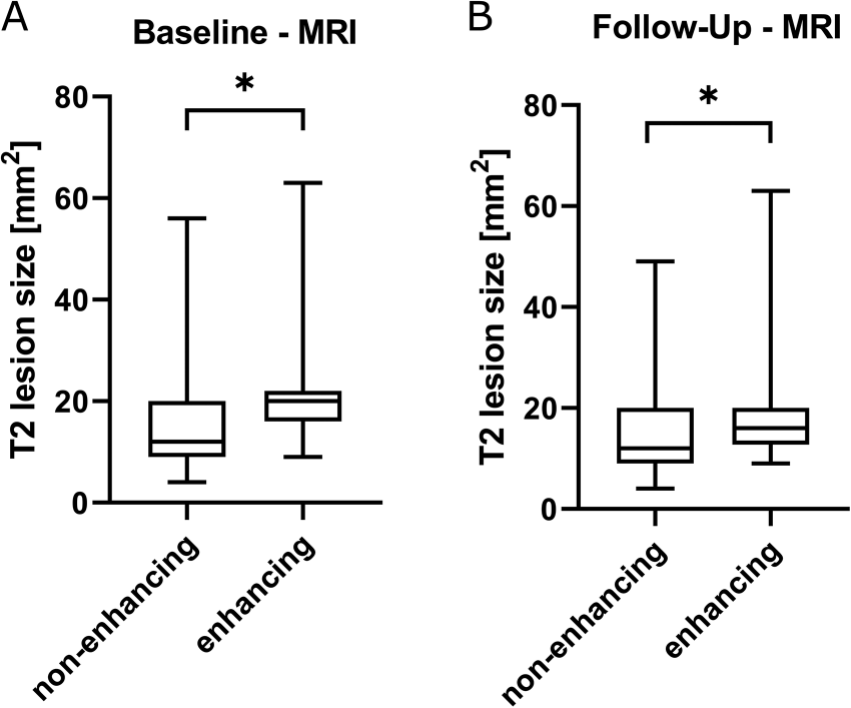
Comparison of baseline and follow-up magnetic resonance imaging (MRI) of multiple sclerosis (MS) patients: enhancing and non-enhancing T2 lesions show significant differences in size on baseline MRI (n = 183; n = 25) and follow-up MRI (n = 135 non-enhancing lesions; n = 44 enhancing lesions; a, b; *p < 0.05). Box shows the 25th and 75th quartiles. Whiskers indicate the minimum and maximum values.

**Figure 2.**
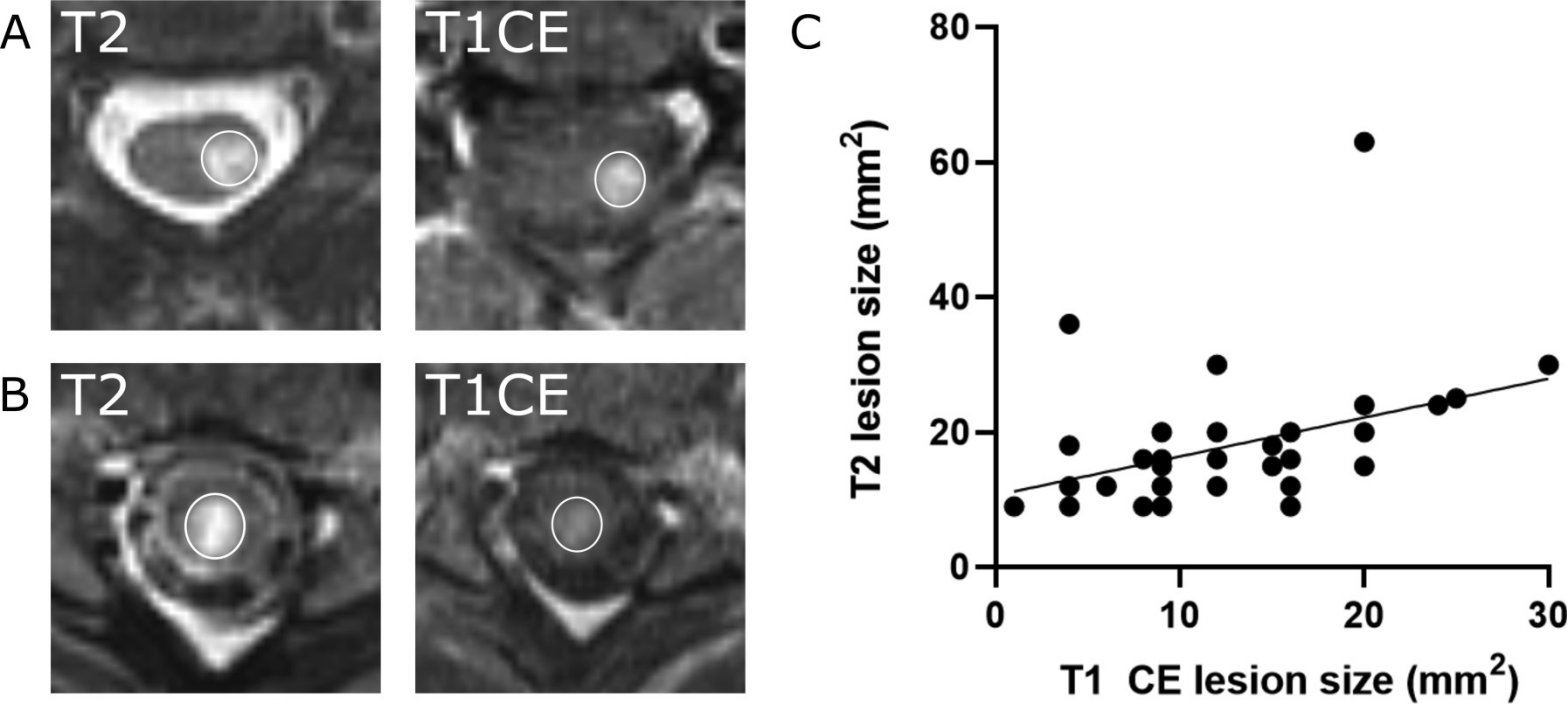
(a and b) T2-weighted (#T2 -w) and T1-weighted (T1-w) contrast-enhanced images. (c) The size of the T2 lesion weakly correlates with the size of the contrast enhancement (CE). Pearson’s correlation analysis (R^2^ = 0.17, **p < 0.01, n = 44 lesions).

We further investigated if the lesion characteristics on T2-w images correlated with lesion enhancement. Non-enhancing new T2 lesions in both baseline and follow-up MRI were significantly smaller in size than lesions that showed enhancement (baseline: mean size 15.7 mm^2^ vs. 20.4 mm^2^, p = 0.03; follow-up: mean size 15.2 mm^2^ vs. 18.5 mm^2^, *p* = 0.04, Figure 1). There was also a very weak, but significant correlation of the T2 lesion size with the size of enhancement (Pearson's correlation coefficient: R^2^ = 0.17, *p* = 0.005, Figure 2).

To assess whether the T2-w lesion size correlates with the size of lesion enhancement as well as with the probability of lesion enhancement, we quantified the maximum area of new T2 lesions with and without enhancement. ROC analysis showed that the T2 lesion size has a poor prognostic rate for lesion enhancement and therefore should not be considered as a predictive factor for Gd enhancement (area under the curve, AUC: 0.65; 95% CI: 0.56−0.73) optimal threshold value: 15 mm^2^, sensitivity: 0.75, specificity: 0.45; *p* = 0.004).

### T2 signal ratio as a correlative marker for Gd enhancement

**Figure 3.**
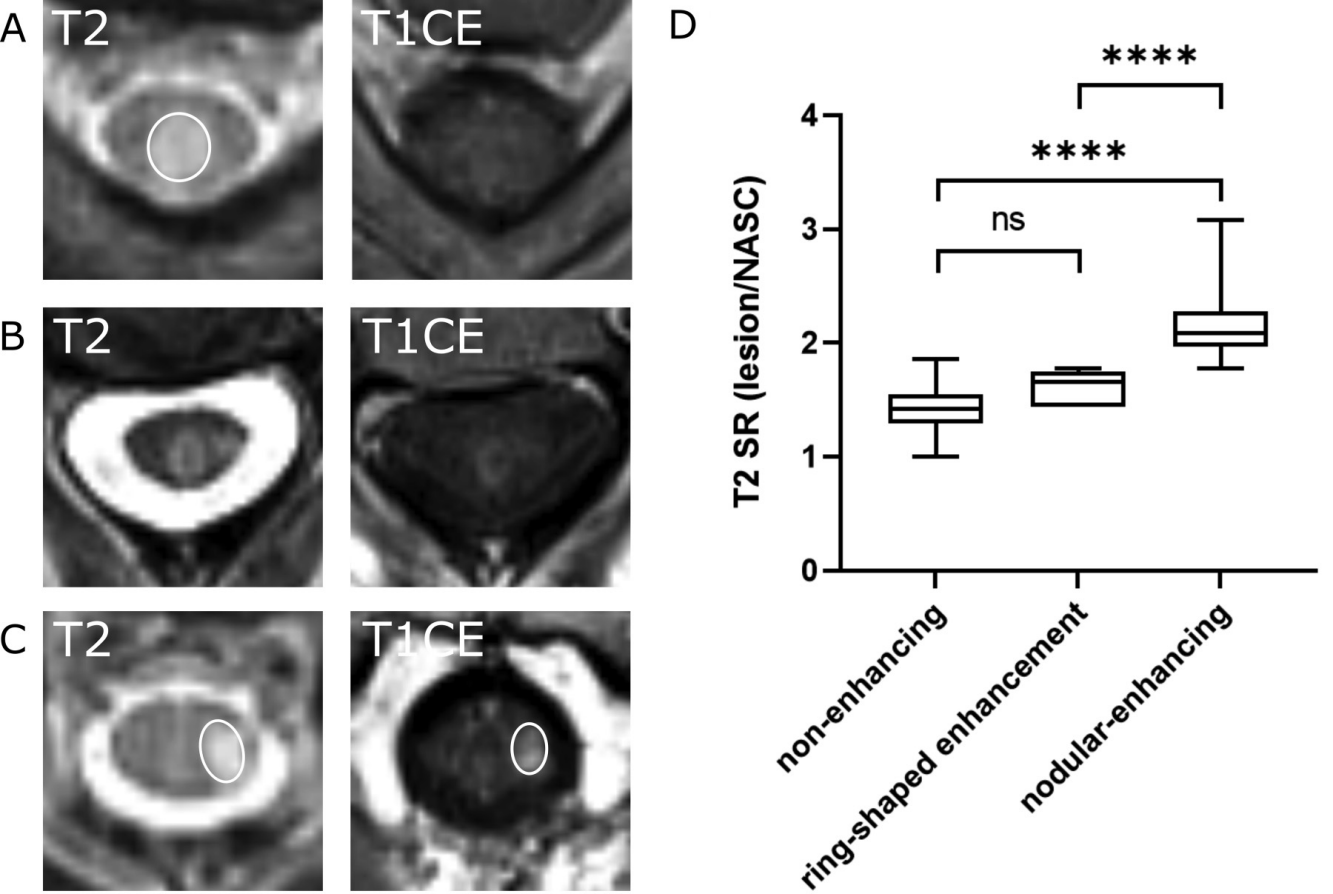
Representative magnetic resosnance imaging (MRI) images of axial T2-weighted (T2-w) and T1-weighted (T1-w) contrast enhancement (CE) images showing different lesion morphologies. (a) T2 lesion without corresponding enhancement and a low T2 signal ratio (SR) of 1.54. (b) Ring-enhancing lesion with a T2 SR of 1.78. (c) T2 lesion with a high T2 SR of 1.93 and clear contrast enhancement (CE). (d) Nodular enhancing lesions (n = 33) showed significantly higher T2 SR than non-enhancing (n = 185, ****p < 0.0001) and ring-shaped enhancing lesions (n = 5****p < 0.0001). Box shows the 25th and 75th quartiles. Whiskers indicate the minimum and maximum values.

**Figure 4.**
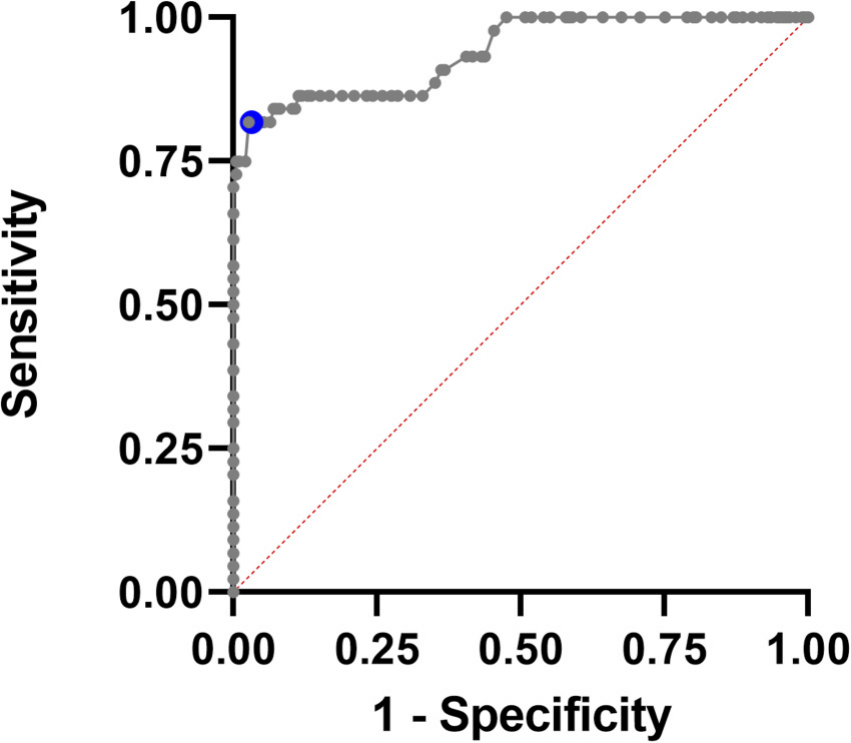
Receiver operating characteristic (ROC) analysis of T2-weighted (T2-w) signal ratio (SR) and likelihood of enhancement. The blue dot indicates the optimal threshold value for the T2 SR of 1.78 (AUC: 0.94, sensitivity: 0.82, specificity: 0.97) to discriminate gadolinium (Gd)-enhancing versus non-enhancing lesions. This indicates that the severity of edema correlates with the probability of enhancement.

We further quantified the T2 SR of Gd-enhancing versus non-enhancing lesions. We found that enhancing lesions showed higher T2 SR than lesions without enhancement (T2 signal_lesion_/T2 signal_normal appearing spinal cord_: 2.0 ± 0.4 vs. 1.4 ± 0.2, ***p < 0.001, Figure 3), indicating that the degree of T2 lesion hyperintensity correlates with the probability of enhancement. As an optimal threshold value to predict lesion enhancement, we calculated a SR of 1.78 (AUC: 0.94, sensitivity: 0.82, specificity: 0.97) for separation of the two groups (Gd-enhancing vs. non-enhancing lesions), as derived from ROC analysis (Figure 4). In a subgroup analysis, we analyzed enhancing lesions that showed ring-shaped CE^20,21^ (11.4% of all enhancing lesions, 5 out of 44). These lesions, however, did not differ significantly in the T2 SR compared to non-enhancing T2 lesions (T2 SR: ring-enhancing lesions: 1.6 ± 0.1 compared to 1.4 ± 0.2, *p* = 0.1).

NASC: Normal appearing spinal cord.

### Correlation of spinal and cerebral disease activity

To determine whether spinal and cerebral disease activity were correlated in time, we evaluated the occurrence of new T2 and CE lesions on spinal and cerebral follow-up MRIs within a time period of ± 3 months. On multivariable logistic regression analysis, patients with spinal T2 lesion activity had a significantly higher risk of new cerebral T2 lesions (relative risk of a lesion at the other site: 9.14, (95% CI: 5.11−16.34); ***p < 0.001), indicating that, in most cases, the relapse affects the entire central nervous system (OR for a concomitant lesion: 51.2, (95% CI: 17.53−149.52); ***p < 0.001). The probability to detect enhancing lesions in the spinal cord or brain when a contrast-enhancing lesion was present in the other location was significantly lower (relative risk: 1.39 (95% CI: 1.11−1.74), OR: 5.11; ***p < 0.001).

## Discussion

Recent studies have questioned the necessity of Gd administration for the imaging follow-up of people with MS showing that Gd contrast administration does not substantially improve disease monitoring.^15,16,20,22–24^ Also, imaging and histopathological assessments demonstrated that linear, and to a lesser degree also macrocyclic, Gd contrast agents can accumulate in the brain parenchyma, although no clinical sequelae have been proven.^11,21^ In our recent study, we identified <5% of cerebral lesions that showed either lesion reactivation (novel enhancement of a preexisting T2 lesion), persistent enhancement or that could only be delineated on CE images but were not discernable on T2/FLAIR images. Interestingly, in spinal MRI, we found no indication of lesion reactivation or persistent enhancement and all CE lesions presented as a new T2 lesion.

The utility of Gd contrast to assess disease activity in MS is a matter of debate.^25–27^ While Gd-administration is required in the setting of diagnosis as well as prior to and one year after the start of a new disease-modifying drug,^28^ it seems mandatory to reconsider the administration of GBCA during follow-up MRI of the clinically stable MS patient.^29^ Given that the probability to miss enhancing lesions with a stable T2 lesion load seems negligible and with the rationale to work as timely and cost-effective as possible, acquisition of CE images can be omitted in routine follow-up.

As an imaging marker to “predict” lesion enhancement we found a small but significant difference in lesion size of enhancing compared to non-enhancing lesions. However, the size of a lesion is not a reliable discriminating factor for lesion enhancement based on our ROC analysis. In contrast, the T2 SR of a given lesion showed a higher sensitivity and specificity (AUC: 0.94) for lesion enhancement.

Furthermore, disease activity in the spinal cord and brain was strongly correlated with a high likelihood of concomitant spinal and cerebral lesion development. This was particularly apparent for new T2 lesions that occurred synchronously at each site, whereas simultaneous contrast-enhancing lesions were less frequent. We conclude that new lesions do not spatially develop in an “either-or” pattern but rather affect the entire central nervous system. This is in line with previous findings reporting a relation between lesion development in the brain and the spinal cord as well as simultaneous CE of cerebral and spinal lesions.^30^

Based on the current study and our previous findings, we propose a “single-shot” MS protocol for the clinically stable MS patient (Table 3). The required MR sequences without the need for Gd administration result in a total scanning time of 23:31 min in our institution (time reduction of 35% when compared to our previous MR protocols that included post-contrast sequences). A shortened, single-session MR protocol may result in higher patient compliance and would be more time and cost-effective.

**Table 3.** “Single shot” multiple sclerosis (MS) protocol.

**Standard MS protocols**		**“Single shot” MS protocol**	
**Recommended Sequences**		**Recommended Sequences**	
**Parameter**	**Duration (min:s)**	**Parameter**	**Duration (min:s)**
**Cerebral**		**Cerebral**	
**FLAIR (3D)**	5:12	**FLAIR (3D)**	5:12
**T2 (TSE, axial)**	2:37	**T2 (TSE, axial)**	2:37
**DWI**	2:48	**DWI**	---
**T1 (MP-RAGE)**	3:47	**T1 (MmP-RAGE)**	3:47
**Total (cerebral)**	**14:24**	**Total (cerebral)**	**11:36**
**Spinal**		**Spinal**	
**T2 (TSE, axial)**	4:51	**T2 (TSE, sagittal)**	4:51
**T2 (TSE, sagittal)**	7:04	**T2 (TSE, axial)**	7:04
**T1 (TSE, sagittal)**	4:22	**T1 (TSE, sagittal)**	---
**T1 (TSE, axial)**	6:03	**T1 (TSE, axial)**	---
**Total (spinal)**	**22:20**	**Total (spinal)**	**11:55**
**Total (cer. and spi.)**	**36:44**	**Total (cer. and spi.)**	**23:31**
**Optional sequences**			
**Cerebral**		**Spinal**	
-**T1-Gd^+^ (PP-RAGE)** - **DTI**		-**T1-Gd^+^ (sagittal)** - **T1-Gd^+^ (axial)** - **STIR (sagittal)**	
**Injection of gadolinium**			
- **Initial diagnosis** - **New cerebral or spinal T2 lesions** - **Clinical relapses**			

FLAIR: fluid-attenuated inversion recovery; MP-RAGE: magnetization-prepared rapid acquisition gradient-echo; TSE: turbo spin echo.

The Magnetic Resonance Imaging in MS (MAGNIMS) consensus guidelines do not recommend spinal cord MRI for routine monitoring and suggest limiting the use of clinical situations with new, unexpected, or unexplained spinal cord symptoms.^31^ In line with previous studies,^32^ a majority of patients with a clinical relapse showed new spinal CE lesions (20 out of 27 patients, 74%). As in our study all CE lesions were discernable as new T2 lesions, both features, when developing newly during follow-up, constitute active disease. Thus, Gd administration would add little information and the use of our proposed “single-shot” protocol seems also justified in patients with clinical spinal cord disease progression.

Limitations of our study include the retrospective, monocentric design with different scan intervals and a variable number of MRI follow-up scans for each patient. Also, most spinal follow-up imaging was performed at least one year apart from baseline MRI, thus persistent enhancement of a given lesion could have been missed. Another limitation is the low number of patients with progressive MS, considering that the clinical course of this MS type is more complex and might be associated with an increased spinal lesion load.^4^ Furthermore, all exams in our study were acquired at 3 T and TSE sequences with a slice thickness of 3 mm were used. Previous studies that compared 3 T and 1.5 T, including multicenter studies, showed that the sensitivity of spinal lesion detection was not adversely affected by higher field strength.^33,34^ Nonetheless, considering that most routinely performed spinal MRIs are performed on devices with a field strength of 1.5 T, a direct comparison between these two field strengths could be performed in future studies to validate our findings. Therefore, whether our findings can be translated to spinal MRI performed at other field strengths and different sequence protocols remains to be investigated.

In general, spinal MRI has lower axial resolution than cerebral MRI and image quality can be hampered by motion and pulsation artifacts, so small CE lesions might be hidden in noise or partial volume, especially at the thoracic level. Furthermore, the number of available spinal MRI scans is smaller compared to our cerebral MRI cohort resulting in smaller subgroups, which decreases statistical power.

In summary, our study shows that patients with a stable spinal T2 lesion load do not benefit from the administration of GBCAs. In contrast to our previous cerebral study, there was no lesion reactivation, persistent enhancement, or lesion that was only detectable after CE. We further show that the T2 SR of a given lesion strongly correlates with CE and could be used as a “predictive marker” for lesion enhancement. In conjunction with previous studies on the limited value of Gd administration in cerebral MS follow-up investigations, we propose a “single-shot” MS protocol to combine cerebral and spinal MRI investigations in one standardized single MR protocol to monitor the clinically stable MS patient.

## Supplementary Material

Supplementary material
