# Supplementary material for: Diagnostic value of gadolinium contrast administration for spinal cord magnetic resonance imaging in multiple sclerosis patients and correlative markers of lesion enhancement
Source: Mult Scler J Exp Transl Clin. 2021 Nov 26;7(4):20552173211047978. doi: 10.1177/20552173211047978 (PMC8637714; doi:10.1177/20552173211047978)
Supplement: Supplementary material [file sj-doc-1-mso-10.1177_20552173211047978.doc]

**MS Journal Appendix for MRI methodology**

| Hardware | |
| --- | --- |
| Field strength | 3 T |
| Manufacturer | Siemens |
| Model | Verio; Prisma; Skyra |
| Coil type  (e.g. head, surface) | spine |
| Number of coil channels | 24 |

| Acquisition sequence | | |
| --- | --- | --- |
| Type  (e.g. FLAIR, DIR, DTI, fMRI) | T2w TSE | |
| Acquisition time | 4:51 | |
| Orientation | **Axial** | |
| Alignment  (e.g. anterior commissure/poster commissure line) | Vertebral column | |
| Voxel size | 0.78 | |
| TR | 4250 | |
| TE | 102 | |
| TI |  | |
| Flip angle | 150 | |
| NEX | 2 | |
| Field of view | 200x200 | |
| Matrix size | 256x256 | |
| Parallel imaging |  | **No** |
| If used, parallel imaging method:  (e.g. SENSE, GRAPPA) |  | |
| Cardiac gating |  | **No** |
| If used, cardiac gating method:  (e.g. PPU or ECG) |  | |
| Contrast enhancement |  | **No** |
| If used, provide name of contrast agent, dose and timing of scan post-contrast administration |  | |
| Other parameters: |  | |

| Acquisition sequence | | |
| --- | --- | --- |
| Type  (e.g. FLAIR, DIR, DTI, fMRI) | T2w TSE | |
| Acquisition time | 3:32 | |
| Orientation | **sagittal** | |
| Alignment  (e.g. anterior commissure/poster commissure line) | Vertebral column | |
| Voxel size | 1 | |
| TR | 3000 | |
| TE | 104 | |
| TI |  | |
| Flip angle | 160 | |
| NEX | 2 | |
| Field of view | 320x320 | |
| Matrix size | 320x320 | |
| Parallel imaging |  | **No** |
| If used, parallel imaging method:  (e.g. SENSE, GRAPPA) |  | |
| Cardiac gating |  | **No** |
| If used, cardiac gating method:  (e.g. PPU or ECG) |  | |
| Contrast enhancement |  | **No** |
| If used, provide name of contrast agent, dose and timing of scan post-contrast administration |  | |
| Other parameters: |  | |

| Acquisition sequence | | |
| --- | --- | --- |
| Type  (e.g. FLAIR, DIR, DTI, fMRI) | T1 TSE | |
| Acquisition time | 6:03 | |
| Orientation | **sagittal** | |
| Alignment  (e.g. anterior commissure/poster commissure line) | Vertebral column | |
| Voxel size | 0.9 | |
| TR | 935 | |
| TE | 9.3 | |
| TI |  | |
| Flip angle | 125 | |
| NEX | 2 | |
| Field of view | 180x180 | |
| Matrix size | 256x256 | |
| Parallel imaging |  | **No** |
| If used, parallel imaging method:  (e.g. SENSE, GRAPPA) |  | |
| Cardiac gating |  | **No** |
| If used, cardiac gating method:  (e.g. PPU or ECG) |  | |
| Contrast enhancement | **Yes** |  |
| If used, provide name of contrast agent, dose and timing of scan post-contrast administration | 0.1 mmol/kg; Dotarem, Guerbet, France | |
| Other parameters: |  | |

| Acquisition sequence | | |
| --- | --- | --- |
| Type  (e.g. FLAIR, DIR, DTI, fMRI) | T1 TSE | |
| Acquisition time | 2:11 | |
| Orientation | **Axial** | |
| Alignment  (e.g. anterior commissure/poster commissure line) | Vertebral column | |
| Voxel size | 0.7 | |
| TR | 600 | |
| TE | 11 | |
| TI |  | |
| Flip angle | 150 | |
| NEX | 1 | |
| Field of view | 320x320 | |
| Matrix size | 307x384 | |
| Parallel imaging |  | **No** |
| If used, parallel imaging method:  (e.g. SENSE, GRAPPA) |  | |
| Cardiac gating |  | **No** |
| If used, cardiac gating method:  (e.g. PPU or ECG) |  | |
| Contrast enhancement | **Yes** |  |
| If used, provide name of contrast agent, dose and timing of scan post-contrast administration | 0.1 mmol/kg; Dotarem, Guerbet, France | |
| Other parameters: |  | |

| Image analysis methods and outputs | |
| --- | --- |
| ***Lesions*** | |
| Type  (e.g. Gd-enhancing, T2-hyperintense, T1-hypointense) | Lesions (Gd-enhancing and T2-hyperintense lesions) |
| Analysis method | Radiological analysis; bidirectional measurement; ROI based-signal intensity quantication |
| Analysis software | GE PACS; Microscoft Excel; PRISM |
| Output measure  (e.g. count or volume [ml]) | mm^2^ |
| ***Tissue volumes*** | |
| Type  (e.g. whole brain, grey matter, white matter, spinal cord) |  |
| Analysis method |  |
| Analysis software |  |
| Output measure  (e.g. absolute tissue volume in ml, tissue volume as a fraction of intracranial volume, percentage change in tissue volumes) |  |
| ***Tissue measures (e.g. MTR, DTI, T1-RT, T2-RT, T2*, T2’, ^1^H-MRS, perfusion, Na)*** | |
| Type  (e.g. whole brain, grey matter, white matter, spinal cord, normal-appearing grey matter or white matter) |  |
| Analysis method |  |
| Analysis software |  |
| Output measure |  |
| ***Other MRI measures (e.g. functional MRI)*** | |
| Type  (e.g. whole brain, grey matter, white matter, spinal cord, normal-appearing grey matter or white matter) |  |
| Analysis method |  |
| Analysis software |  |
| Output measure |  |

**Other analysis details:**
